# Comparison of clinical features and outcomes in COVID-19 and influenza pneumonia patients requiring intensive care unit admission

**DOI:** 10.1007/s15010-021-01624-7

**Published:** 2021-05-26

**Authors:** A. Oliva, G. Ceccarelli, C. Borrazzo, M. Ridolfi, G. D.’Ettorre, F. Alessandri, F. Ruberto, F. Pugliese, G. M. Raponi, A. Russo, A. Falletta, C. M. Mastroianni, M. Venditti

**Affiliations:** 1 Department of Public Health and Infectious Diseases, Sapienza University of Rome, Piazzale Aldo Moro 5, 00185 Rome, Italy; 2 Department of Anesthesia and Critical Care Medicine, Sapienza University of Rome, Policlinico Umberto I, Rome, Italy

**Keywords:** COVID-19, Influenza, SARS-CoV-2, Intensive care unit, Invasive pulmonary aspergillosis, Superinfections, Thrombotic events

## Abstract

**Background:**

Little is known in distinguishing clinical features and outcomes between coronavirus disease-19 (COVID-19) and influenza (FLU).

**Materials/methods:**

Retrospective, single-centre study including patients with COVID-19 or FLU pneumonia admitted to the Intensive care Unit (ICU) of Policlinico Umberto I (Rome). Aims were: (1) to assess clinical features and differences of patients with COVID-19 and FLU, (2) to identify clinical and/or laboratory factors associated with FLU or COVID-19 and (3) to evaluate 30-day mortality, bacterial superinfections, thrombotic events and invasive pulmonary aspergillosis (IPA) in patients with FLU versus COVID-19.

**Results:**

Overall, 74 patients were included (19, 25.7%, FLU and 55, 74.3%, COVID-19), median age 67 years (58–76). COVID-19 patients were more male (*p* = 0.013), with a lower percentage of COPD (Chronic Obstructive Pulmonary Disease) and chronic kidney disease (CKD) (*p* = 0.001 and *p* = 0.037, respectively) than FLU. SOFA score was higher (*p* = 0.020) and lymphocytes were significantly lower in FLU than in COVID-19 [395.5 *vs* 770.0 cells/mmc, *p* = 0.005]. At multivariable analysis, male sex (OR 6.1, *p* < 0.002), age > 65 years (OR 2.4, *p* = 0.024) and lymphocyte count > 725 cells/mmc at ICU admission (OR 5.1, p = 0.024) were significantly associated with COVID-19, whereas CKD and COPD were associated with FLU (OR 0.1 and OR 0.16, *p* = 0.020 and *p* < 0.001, respectively). No differences in mortality, bacterial superinfections and thrombotic events were observed, whereas IPA was mostly associated with FLU (31.5% vs 3.6%, *p* = 0.0029).

**Conclusions:**

In critically ill patients, male sex, age > 65 years and lymphocytes > 725 cells/mmc are related to COVID-19. FLU is associated with a significantly higher risk of IPA than COVID-19.

## Introduction

The novel β-coronavirus, named Severe Acute Respiratory Syndrome Coronavirus 2 (SARS-CoV-2), due to the similarity with the virus that caused the SARS outbreak in 2002–2004, emerged in Wuhan, China, in December 2019 and has rapidly spread through the world causing the ongoing pandemic [1]. At the time of writing, it has infected over 112 million people and resulted in more than 2.4 million deaths worldwide, especially in patients admitted to Intensive Care Unit (ICU) or those developing Acute Respiratory Distress Syndrome (ARDS) [2].

As a consequence, the adopted interventions such as lockdown and social limitation contributed also to the reduction of other respiratory viral infections including influenza [3].

SARS-CoV-2 and influenza viruses share similar aspects, from their ease of transmission from person-to-person through the respiratory droplet route to their clinical presentation. In fact, both viruses can cause acute respiratory failure that might require hospitalization in ICU, might be complicated by bacterial and fungal superinfections and might predispose subjects to the development of thrombosis [4–11].

However, little is known in distinguishing clinical features and outcomes in critically ill patients with coronavirus disease-19 (COVID-19) and influenza (FLU) requiring ICU admission.

The development of bacterial and fungal superinfections occurs likely due to severe pulmonary inflammation, compromised pulmonary defenses, ventilator dependence, and the receipt of immunosuppressive drugs, all conditions present during FLU and COVID-19 [4, 5, 12]. In particular, while the development of influenza-associated pulmonary aspergillosis (IAPA) is a well-recognized condition, the occurrence of invasive pulmonary aspergillosis (IPA) as a consequence of COVID-19, although increasingly reported, is still a matter of debate [13–16]. Several reports of COVID-19-associated pulmonary aspergillosis (CAPA) have been described so far; however, the real incidence of this condition in critically ill patients is still unknown, ranging from 3.3 to 33.3%, and comprehensive data are lacking [17–27].

Pulmonary thrombosis and vasculitis occur in subgroups of adult patients with severe influenza, but this phenomenon seemed to be highly prevalent in patients with COVID-19 and has been considered also responsible for unfavorable outcomes [9, 28].

Therefore, based on these premises, the aims of the study were: (1) to assess the clinical features and differences of critically ill patients with COVID-19 and FLU, (2) to identify clinical and/or laboratory factors associated with FLU or COVID-19 and (3) to evaluate the clinical outcomes in critically ill patients with FLU and COVID-19, expressed as 30-day mortality, development of bacterial superinfections, documented thrombotic events and IPA.

## Materials and method

### Study population

A retrospective, single-center study including all adult patients with diagnosis of COVID-19 or severe FLU requiring ICU admission and hospitalized at Azienda Policlinico Umberto I, “Sapienza” University of Rome was performed. Patients with FLU and COVID-19 pneumonia were hospitalized from January to December 2019 and from March to September 2020, respectively. During the 2020 winter season, our hospital was exclusively dedicated to the cure of COVID-19 patients; therefore, no patients with FLU in 2020 could have been included in the study.

Nasopharyngeal swab samples or bronchoalveolar fluid were collected and SARS-CoV-2 or influenza RNA were detected using RT-PCR (RealStar SARS-CoV2 RT-PCR, Altona Diagnostics). For each subject, demographic, laboratory, clinical and radiological data were collected and recorded anonymously in an electronic database. The study was approved by the local Ethics Committee (ID Prot. 109/2020).

As for local protocol, screening for multi-drug-resistant bacteria (i.e., methicillin-resistant *Staphylococcus aureus*, MRSA, or carbapenem resistant Gram-negative bacilli) colonization was systematically performed by means of rectal swab and lower respiratory tract cultures. The clinical approach to bacterial and fungal superinfection was managed by two dedicated ID physicians (GC and MV).

**Fig. 1:**
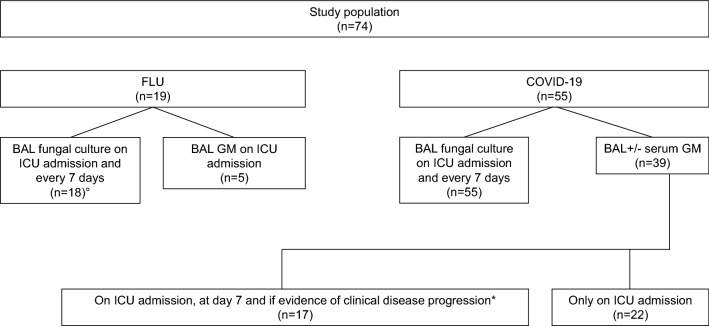
Flowchart of the study. *FLU* influenza, *COVID-19* Coronavirus Disease 19, *BAL* Bronchoalveolar lavage, *GM* galactomannan, *ICU* Intensive Care Unit. **a** according to the algorithm proposed by Bartoletti et al. [17]. **b** in case#6 BAL was not performed and invasive pulmonary aspergillosis (IPA) was proven at autopsy

In 18 out of 19 FLU and all COVID-19 patients, BAL with fungal cultures was systematically performed at ICU admission and every 7 days. In FLU patients, BAL GM was performed either at ICU admission or subsequently based on clinical suspicion of IPA superinfection: thus, it was eventually done in 5 patients (Fig. 1). On the other hand, in the COVID-19 cohort, BAL GM was performed in 39 out of 55 patients (70.9%). As shown in Fig. 1, during the first period of the study (March–May 2020), BAL for GM was collected only on ICU admission in 22 out of 39 COVID-19 patients (56.4%). Based on the increasing numbers of CAPA reports accumulating in the literature [17–27], in the second period of the study (June 2020–September 2020), BAL for GM was collected also at day 7 (± 2 days) and in instances of pulmonary disease progression.

Fungal cultures were incubated for 7 days at 30 °C on Sabouraud selective media. GM test in serum and BAL was performed according to manufacturer’s instructions (Platelia Aspergillus EIA, Bio-Rad).

Clinical outcomes included 30-day mortality, bacterial superinfections, thrombotic/embolic events and the development of IPA.

### Definitions

As for COVID-19, definition of pneumonia or severe pneumonia was based on the WHO interim guidance and included clinical signs of pneumonia (fever, cough, dyspnea, fast breathing) with or without signs of severe pneumonia such as respiratory rate > 30 breaths/min, severe respiratory distress, or SpO2 < 90% on room air [29].

Thrombotic/embolic events were defined as the appearance of new ischemic/embolic events diagnosed as follows: (1) pulmonary thrombo-embolism by lung computed tomography (CT) scan (2) myocardial infarction by EKG changes associated with enhanced markers of cell necrosis (3) acute brain ischemia by onset of new focal neurological signs and symptoms and confirmed, whenever possible, by magnetic resonance or CT imaging and (4) acute limb ischemia [5].

Bacterial superinfections were diagnosed and defined in accordance to guidelines [4].

IPA following FLU (IAPA) was defined in accordance with the recent expert case definition and modified AspICU algorithm [13, 14]. IPA following COVID-19 was defined according to the modified AspICU algorithm [13] and/or the recently proposed definition of CAPA: COVID-19 positive ICU patients with pulmonary infiltrates (entry criterion) who had at least one of the following (a) serum galattomannan (GM) index > 0.5 or (b) bronchoalveolar (BAL) GM index > 1.0 or (c) positive *Aspergillus* spp BAL culture or (d) cavitating infiltrate (not attributed to another cause) in the area of the pulmonary infiltrate [16].

### Statistical analysis

All statistical data were analyzed using the Statistical Package for the Social Sciences (SPSS) version 22. The data, unless otherwise stated, were given as means with standard deviation (SD), minimum and maximum or medians with Interquartile ranges (IQR, 25th–75th) for continuous variables and as simple frequencies, proportions and relative percentages for dichotomous variables. Student’s *t* test or Mann–Whitney U test were used for comparison means or medians in the univariate analyses. Between-group comparisons of dichotomous variables were conducted by using chi-square tests (or Fisher’s exact test) and odds ratios (ORs) with 95% confidence intervals (95% CIs). OR values < 1 indicates association with FLU, whereas ORs > 1 indicates association with COVID-19. For lymphocytes, an absolute count of 725 cells/mmc was used [30]. *p* value analyses were two-sided and a *p* value of less than 0.05 was considered statistically significant.

## Results

### Study population

**Fig. 2:**
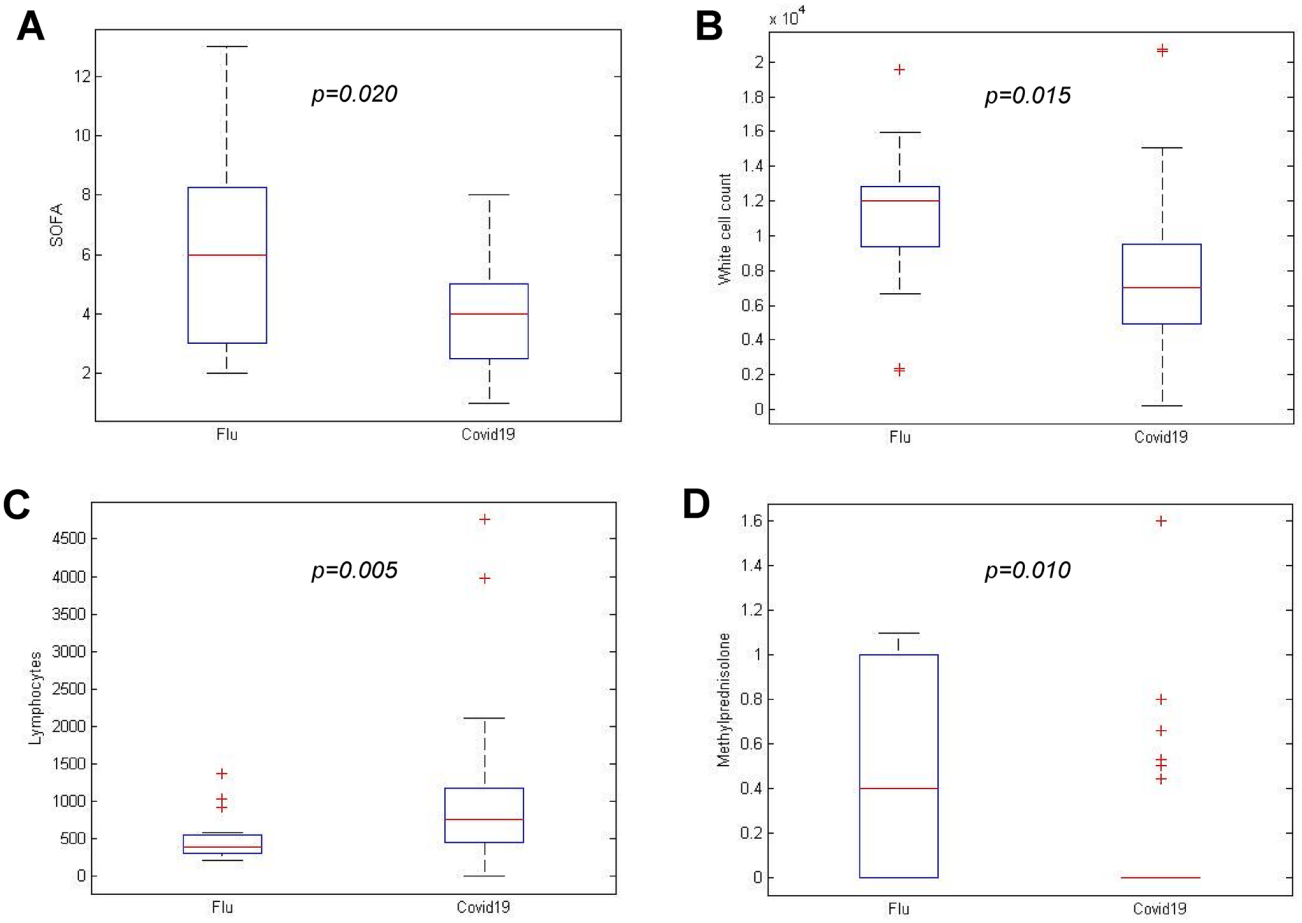
Differences in SOFA score (**a**), White Blood Cells (**b**), Lymphocyte (**c**) absolute count and daily dosage of methylprednisolone (mg/kg/die) (**d**) between patients with influenza (FLU) and coronavirus disease-19 (COVID-19). *SOFA* Sequential Organ Failure Assessment

Overall, 74 critically ill patients were included in the study (19, 25.7%, with FLU and 55, 74.3%, with COVID-19, respectively), median age was 67 years (58–76) (71.5 *vs* 67 for FLU and COVID-19 subjects, respectively, *p* = 0.807) (Table 1). All but one in the FLU group were community-acquired infections. Compared with FLU ones, patients with COVID-19 were more frequently male (44, 80%, *vs* 9, 47%, *p* = 0.013). Overall, no differences in the Charlson Comorbidity Index were found between the 2 groups (*p* = 0.102), with a lower percentage of smoke, COPD (Chronic Obstructive Pulmonary Disease) and chronic kidney disease (CKD) in patients with COVID-19 than those with FLU (25% *vs* 63.1%, *p* = 0.004, 20% *vs* 57.8%, *p* = 0.003 and 4% *vs* 26.3%, *p* = 0.010, respectively). At ICU admission, severity of infection was higher in FLU than in COVID-19 patients (median SOFA score 6 *vs* 4, *p* = 0.020) (Fig. 2a). White Blood Cells (WBCs) were higher in FLU than in COVID-19 [12010 (9820–12,700) *vs* 7030 (4927.5–9510) cells/mmc, *p* = 0.015]; however, lymphocytes absolute count was significantly lower in FLU patients [395.5 (316–541) *vs* 770.0 (465–1165) cells/mmc, *p* = 0.005] (Fig. 2b, c).

**Table 1:** General characteristics of study population

Parameter	Total	FLU	COVID-19	*p value*
	*N*=74	*N*=19	*N*=55	
Male Sex, *n* (%)	52 (71)	9 (47)	44 (80)	0.013
Age, y, median (IQR)	67 (58–76)	71.5 (59.5–76.75)	67 (58–75)	0.807
WBC, cells/µL	8550	12010	7030	0.015
median (IQR)	(5260–11600)	(9820–12700)	(4927–9510)	
Neutrophils, cells/µL	7094	10665	5705	0.001
median (IQR)	(4070–10158)	(9345–11812)	(3677–8332)	
Lymphocytes, cells/µL	610 (370–1036)	395.5 (316–541)	770 (465–1165)	0.005
median (IQR)				
CRP, mg/dL	12.67	50.9	12	0.113
median (IQR)	(5.37–28.47)	(22.6–54.2)	(5.03–25.45)	
Smoke, *n* (%)	26 (35.1)	12 (63.15)	14 (25)	0.004
CCI, median (IQR)	3 (2–5)	4 (2–7)	3 (1.5–4.5)	0.102
SOFA, median (IQR)	4 (3–5)	6 (3–8)	4 (2.75–5)	0.02
≥ 1 comorbidity, *n* (%)	56 (75.6)	16 (84)	40 (73)	0.37
COPD, *n* (%)	22 (29.7)	11 (57.8)	11 (20)	0.003
CKD, *n* (%)	7 (9.4)	5 (26.3)	2 (4)	0.01
Hemodialysis, *n* (%)	23 (31)	4 (21)	19 (35)	0.39
ECMO, *n* (%)	8 (10.8)	3 (15.7)	5 (9)	0.415
Hypertension, *n* (%)	36 (48.6)	7 (36.8)	29 (53)	0.29
CAD, *n* (%)	21 (28.3)	7 (36.8)	14 (25)	0.38
Cirrhosis, *n* (%)	0 (0)	0 (0)	0 (0)	–
Diabetes mellitus, *n* (%)	22 (29.7)	7 (36.8)	15 (27)	0.56
Neurological disorders, *n* (%)	3 (4)	1 (5.2)	2 (4)	0.9
Cancer, *n* (%)	5 (6.7)	1 (5.2)	4 (7)	0.9
Corticosteroids, *n* (%)	18 (24.3)	5 (26.3)	13 (24)	0.9
Dosage of methylprednisolone, mg/kg/die, median (IQR)	0 (0–0.4)	0.4 (0–1)	0 (0–0)	0.01
Use of broad spectrum antibiotics, *n* (%)	64 (86.4)	12 (63.1)	51 (92.7)	0.0048
Length of hospitalization, days, median (IQR)	22 (15.5–38.5)	21 (12–37)	23 (17.5–36.5)	0.678
Pulmonary super-infections, *n* (%)	31 (73.8)	11 (57.8)	20 (36)	0.11
MRSA	2 (6.4)	0 (0)	2 (10)	0.52
KPC	1 (3.3)	0 (0)	1 (5)	0.9
OXA-48	3 (9.6)	0 (0)	3 (15)	0.53
*Acinetobacter* *baumanii*	9 (29.1)	4 (36.4)	5 (25)	0.68
* Pseudomonas* *aeruginosa*	10 (32.3)	2 (18.2)	8 (40)	0.26
Others^*^	6 (19.3)	5 (45.4)	1 (5)	0.01
BSI, *n* (%)	31 (41.8)	8 (42.1)	23 (42)	0.589
Microorganisms of BSI°, *n* (%)	38 (100)	10 (26.3)	28 (73.7)	
MRSA	1 (2.6)	0 (0)	1 (3.5)	–
KPC	6 (15.7)	3 (30)	3 (10.7)	0.31
OXA-48	1 (2.6)	0 (0)	1 (3.5)	–
*Acinetobacter* *baumanii*	5 (13.1)	0 (0)	5 (17.8)	0.29
*Pseudomonas* *aeruginosa*	1 (2.6)	1 (10)	0 (0)	–
* Candida spp*	4 (10.5)	1 (10)	3 (10.7)	0.9
CoNS	13 (34.2)	3 (30)	10 (35.7)	0.9
* Enterococcus spp*	3 (7.8)	1 (10)	2 (7.1)	0.9
*Enterobacterales*	4 (10.5)	1 (10)	3 (10.7)	0.9
NO KPC NO OXA-48				
MDR colonization, *n* (%)	20 (27)	7 (36.8)	13 (24)	0.564
KPC	7 (9.4)	4 (21)	3 (5)	0.06
OXA-48	4 (5.4)	0 (0)	4 (7)	0.252
*Acinetobacter baumanii*	9 (12.1)	1 (5.1)	8 (15)	0.42
Pulmonary aspergillosis, *n* (%)	8 (10.8)	6 (31.5)	2 (3.6)	0.0029
Thrombotic events, *n* (%)	15 (20)	3 (15.7)	12 (21.8)	0.521
30-day mortality, *n* (%)	50 (67.5)	12 (63.2)	38 (69)	0.77

*COVID-19* coronavirus disease-19; *FLU* influenza; *IQR* interquartile range; *WBC* white blood cells; *CRP* C-reactive protein; *CCI* charlson comorbidity index; *SOFA* sequential organ failure assessment; *COPD* chronic obstructive pulmonary disease; ιιιxygenation; *CAD* coronary artery disease; *MRSA* methicillin-resistant *Staphylococcus aureus*; *KPC* *Klebsiella pneumoniae* carbapenemase; *BSI* bloodstream infection; *CoNS* coagulase negative *Staphylococci*; *MDR* multi-drug resistance.

^a^Others include: *Streptococcus pneumoniae* (*n* = 1), *Stenotrophomonas maltophilia* (*n* = 2), *Enterobacterales* other than KPC and OXA-48 (*n* = 3)

^b^Number of microorganisms causing BSI is higher than the number of patients with BSI

Corticosteroid use did not differ between the two groups (26.3% *vs* 24%, *p* = 0.9), however, the dosage of corticosteroids (methylprednisolone, mg/kg/die) was higher in FLU patients (*p* = 0.010) (Fig. 2, Panel D). Patients with COVID-19 received broad-spectrum antibiotics more frequently than patients with FLU (92.7% *vs* 63.1%, *p* = 0.0048); in detail, teicoplanin and piperacillin/tazobactam were commonly used in COVID-19 critically ill patients (81.8% *vs* 0% and 67.2% *vs* 17%, *p* < 0.0001 and* p* = 0.003, respectively) due to a predefined protocol [31–33]. Patients with COVID-19 and FLU had the same length of hospitalization in the ICU [21 (12–37) *vs* 23 (17.5–36.5) days, *p* = 0.672, respectively].

### Comparison of clinical outcomes

**Fig. 3:**
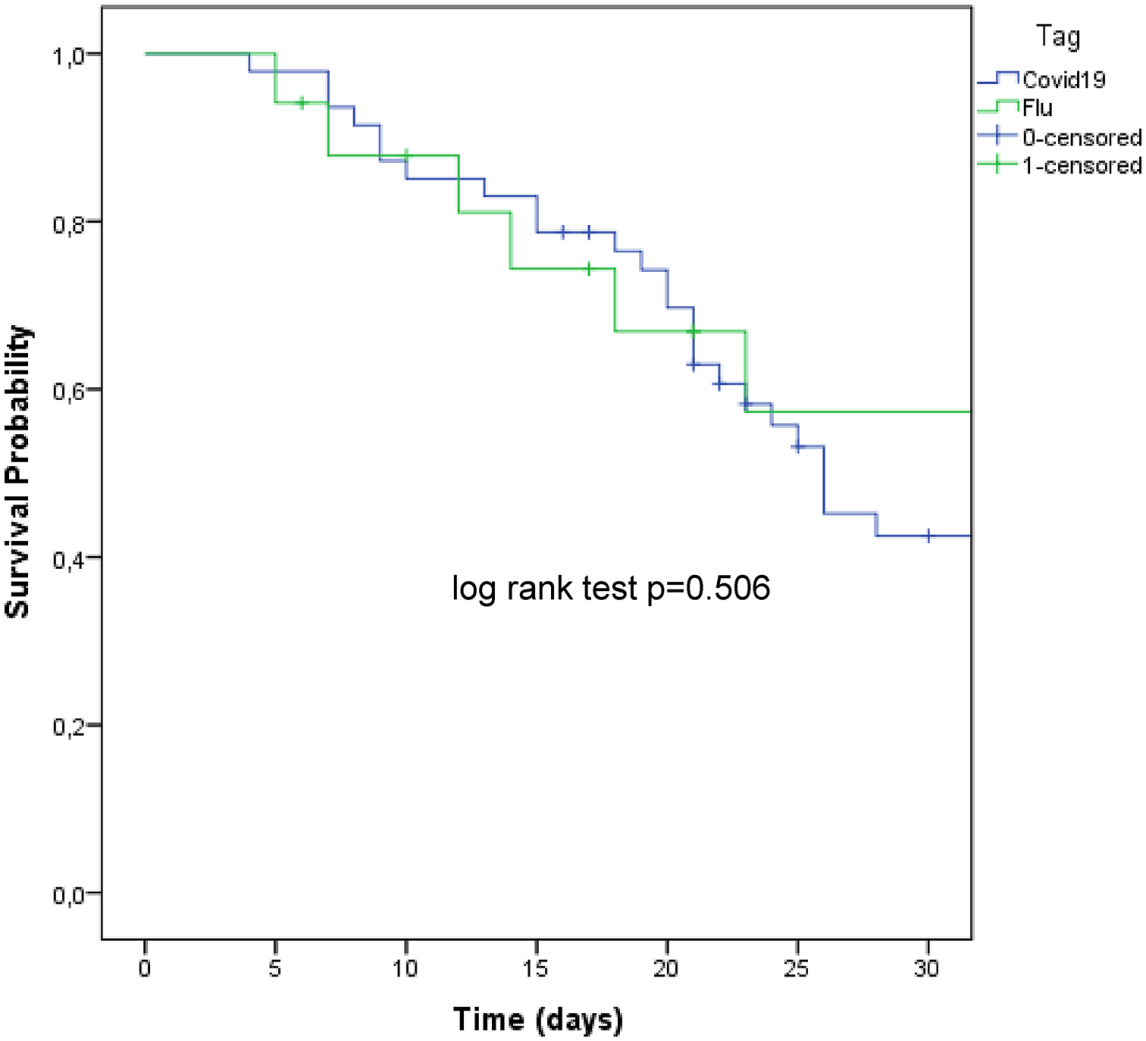
Cumulative survival probability in patients with influenza (FLU) and coronavirus disease-19 (COVID-19)

Overall, 30-day mortality did not differ between the two groups (63.1% *vs* 69% for FLU and COVID-19, respectively, *p* = 0.77) (Fig. 3), as well as the occurrence of bacterial pulmonary superinfections, which, however, tended to be more frequent in FLU (57.8% *vs* 36%, *p* = 0.11). With regard to the isolates causing respiratory superinfections, *Acinetobacter baumannii* was more frequently observed in FLU patients, whereas *Pseudomonas aeruginosa* was more frequent in COVID-19 and OXA-48 carbapenemase-producing *Klebsiella pneumoniae* was found only in COVID-19 subjects. The latter finding was confirmed by the rate of OXA-48 rectal colonization in COVID-19 patients, probably representing a small outbreak within the ICU, which was further limited with appropriate infection control measures [34]. The rate of bloodstream infections was similar, with 8 (42.1%) and 23 (42%) events in FLU and COVID-19 subjects, respectively (*p* = 0.589). Again, no difference among causative agents was found.

Documented thrombotic events were higher in patients with COVID-19 (12/55, 21.8%) compared to FLU group (3/19, 15.7%), in the absence of statistical significance (*p* = 0.521).

BAL GM was performed in 18 out of the 19 FLU patients. Conversely, in patients with COVID-19, BAL GM was performed in 39 patients (70.9%), only on ICU admission during the first period of pandemic (March–May 2020) and on ICU admission, at day 7 and if the patient showed evidence of clinical disease progression thereafter (Fig. 1).

IPA was significantly associated with FLU with only two cases of CAPA detected over the study period (6/19, 31.5% vs 2/55, 3.6%, *p* = 0.0029). BAL GM was positive in 4/5 (80%) patients with IAPA, BAL culture detected *Aspergillus fumigatus* in one patients and serum GM, when performed, was negative. Autopsy proved IPA in one patient with FLU, with growth of *Aspergillus fumigatus*. In patients with COVID-19, BAL cultures were negative, whereas BAL GM was positive in two patients. According to the modified AspICU algorithm, FLU patients had one proven and four putative IPA, whereas no patients with COVID-19 had proven or putative IPA (Table2).

**Table 2:** Characteristics of COVID-19 patients with IAPA (*n* = 6) and CAPA (*n* = 2)

Cases	Age, Sex	Comorbidities	Lymphocytes	BAL GM/*Aspergillus* spp growth in BAL culture	Modified AspICU^a^	Radiological findings	Antifungal therapy	Outcome
IAPA (influenza type)								
Case#1 (H1N1)	58, M	CAD	304	1/no growth	Putative	X-ray (diffuse bilateral interstitial infiltrates)	ISA	Died
Case#2 (A)	48, F	COPD	320	3.6/no growth	Putative	CT (AIR with bud in tree lesions + ANGIO with multiple mycetomas)	VOR	Died
Case#3 (H1N1)	71, M	COPD, CKD	300	3.2/no growth	Putative	CT (ANGIO with nodules and mixed alveolar and GG lesions)	VOR	Died
Case#4 (H1N1)	85, F	COPD	274	NP^b^/growth	Putative	CT (ANGIO with multiple mycetomas)	ISA	Died
Case#5 (A)	66, M	COPD	210	> 2.4^c^/no growth	Putative	CT (ANGIO with mycetomas and mixed alveolar and GG lesions)	ISA	Survived
Case#6 (B)	59, M	None	270	NP/NP	Proven*	CT (diffuse bilateral interstitial infiltrates and crazy paving)	CAS	Died
CAPA								
Case#1	39, M	Obesity	1050	> 2.3/no growth	–	CT (Multiple bilateral GG and crazy paving lesions)**	No	Survived
Case#2	58, M	None	1150	> 2.3/no growth	–	CT (Multiple bilateral GG and crazy paving lesions)**	No	Survived

*IAPA* Influenza Associated Pulmonary Aspergillosis, *CAPA* COVID-19 Associated Pulmonary Aspergillosis, *BAL* bronchoalveolar lavage, *SER* serum, *CAD* coronary artery disease, *COPD* chronic obstructive pulmonary disease, *CKD* chronic kidney disease, *NP* not performed, *CT* computed tomography, *GM* galactomannan, *AIR* airway-invasive aspergillosis [41], *ANGIO* angio-invasive aspergillosis [50], *GG* ground glass, *ISA* isavuconazole, *VOR* voriconazole, *CAS* caspofungin.

^a^Modified AspICU was defined in accordance to [13]

^b^serum GM:0.5

^c^BAL GM dropped to 0.9 under therapy

*diagnosis of aspergillosis was confirmed at autopsy with *Aspergillus fumigatus* growth, CAS was started empirically;

**: radiological examinations were consistent with COVID-19 pneumonia

At the multivariable analysis, male sex (OR 6.1, *p* < 0.002), age > 65 years (OR 2.4, *p* = 0.024) and lymphocyte count > 725 cells/mmc at ICU admission (OR 5.1, *p* = 0.024) were significantly associated with COVID-19, whereas the presence of CKD and COPD were associated with FLU (OR 0.1 and OR 0.16, *p* = 0.020 and *p* < 0.001, respectively). Of note, IPA was significantly associated with FLU (OR 0.02, *p* = 0.011) (Table 3).

**Table 3:** Multivariable analysis of factors associated with COVID-19 (OR  >  1) or FLU (OR  < 1)

Parameter	OR	95% CI	*p *value
Male sex	6.1	1.9–18.9	0.002
Age > 65 years	2.4	1.1–6.6	0.024
Lymphocytes > 725/µL	5.1	1.2–21.3	0.024
Corticosteroids	1.3	0.4–4.6	0.631
Dosage of methylprednisolone (mg/kg/die)	0.3	0.01–1.1	0.432
SOFA	0.46	0.1–1.7	0.25
COPD	0.16	0.05–0.5	< 0.001
CKD	0.1	0.01–0.6	0.020
Pulmonary aspergillosis	0.02	0.001–0.42	0.011

*COVID-19* coronavirus disease-19, *FLU* influenza, *SOFA* sequential organ failure assessment, *COPD* chronic obstructive pulmonary disease, *CKD* chronic kidney disease

## Discussion

Symptoms of COVID-19 appear very similar to FLU, especially in the early phase of infection, and therefore, FLU represents an optimal comparator for COVID-19. To date, clinical features and outcomes in critically ill patients with FLU and COVID-19 are not widely described [35–38]. Therefore, in the present study, we contributed to the comparison between FLU and COVID-19 and we found that in critically ill patients requiring ICU admission male sex, age > 65 and higher lymphocytes counts were associated with COVID-19, whereas CKD and COPD were related to FLU, respectively.

Male gender has been already linked to more severe symptoms, higher mortality rate and a more prolonged viral shedding than female in COVID-19 [39]. Our data suggest that gender per se may predispose males to COVID-19, possibly related to the effect of gender on viral receptor expression [40]. Elderly was another independent factor associated more with COVID-19 than with FLU, confirming the literature data available so far, which recognize age as a risk factor for severe COVID-19 and mortality [40]. In contrast, elder subjects were more frequently vaccinated for FLU, with consequent possible lower predisposition to this infection.

COPD was associated more with FLU than with COVID-19, suggesting that the presence of lung damage from a pre-existing condition might be per se necessary for ICU admission in the case of FLU but not in the case of COVID-19.

As far as the clinically relevant outcomes are concerned, no differences in mortality rate, bacterial superinfections and thrombotic events were found. Conversely, IPA was significantly associated with FLU. During the study period, only two cases of CAPA were detected, with non-specific radiological findings and, most important, with recovery even in the absence of antifungal therapy (Table 2). In contrast, patients with IAPA presented with typical air and/or angio-invasive patterns on radiological examination [41] in all but one patients and had poor outcomes (5/6 death, 83.3%) despite antifungal therapy.

Of note, FLU subjects had significantly lower lymphocytes counts than COVID-19 ones (Table 2), possibly explaining the observed higher rate of IAPA than CAPA. As a matter of fact, while lymphocytopenia has been identified as a potential risk factor for *Aspergillus spp* pulmonary superinfection following severe FLU [30], the exact role of lymphocyte count in the pathogenesis of IPA following COVID-19 is still unknown and, in our opinion, deserves additional investigations.

Although IAPA is a well-recognized clinical entity, data regarding the association between IPA and COVID-19 are still conflicting [1–27, 42–46]. Overall, we found a lower prevalence of CAPA (3.6%) than that reported so far, which ranged from 3.3 to 33.3% of ICU subjects with COVID-19, with a recent review of the literature reporting a total of 93 ICU patients who developed IPA after a mean ICU stay of 7 days [27]. Despite this variability, the results of autopsy studies are worth of mention since IPA was a frequent cause of death in COVID-19 patients who eventually developed ARDS [47]. On the other hand, Razazi et al. found a very low prevalence of IPA (diagnosed according to the AspICU algorithm) in COVID-19 patients with ARDS (2/90, 2%) compared to 12/82 (14.6%) in patients with ARDS caused by other viruses [48].

The observed differences in CAPA prevalence might be explained by the difficulty in recognizing this emerging condition, the possible absence of specific radiological patterns and the variability of diagnostic definitions [46]. Last, but not least, it is well known that building-work activities inside or near the hospital allow dust contamination and fungal spores' dissemination and, therefore, might explain differences in IPA prevalence among studies [49]. However, it should be acknowledged that IPA following COVID-19 has been associated to mortality rates higher than 50% [17]. As a consequence, physicians should anyway consider IPA as a possible cause of pneumonia progression in COVID-19 patients and include this potentially fatal complication in the differential diagnosis even in the absence of typical radiological findings.

In the present study, bacterial superinfections were a common finding in both FLU and COVID-19, with a prevalence of Gram-negative microorganisms. These findings are in line with the recent literature date, which showed that bacterial superinfections might complicate up to half of COVID-19 patients admitted to ICU, with a high case fatality rate [4]. However, similarly to what observed for IPA, rates of bacterial superinfections varied widely among the published studies [5, 6]. Therefore, we could speculate that the real prevalence of bacterial and fungal superinfections in COVID-19 critically ill patients is still unknown and may be influenced by the different local epidemiology as well as by the different therapeutic approaches.

Both FLU and COVID-19 are associated with severe cardiovascular and cardiorespiratory complications, mainly due to the occurrence of thrombotic events [7, 9, 28, 50]. Although in the absence of statistical significance, we found a higher occurrence of thrombotic events in patients with COVID-19 than with FLU, confirming that coagulopathy represents a crucial aspect of this condition, possibly driven by the activation of NADPH oxidase and by an excessive harmful pro-inflammatory response [28].

The present investigation has several limitations, which should be taken into consideration. First, the retrospective and single-center nature of the study with the consequent low number of included patients. Then, the search for IPA changed during the study period, a fact that might have possibly underestimate the real prevalence of this condition, which was, however, very low even after the implementation of a more aggressive diagnostic approach. Third, patients with FLU and COVID-19 belonged to different periods since during the 2020 our hospital was exclusively dedicated to COVID-19 patients.

## Conclusion

In critically ill patients, male sex, age > 65 years and lymphocytes absolute count > 725 cells/mmc are related to COVID-19, whereas COPD and CKD are more typical of FLU. In our population, FLU was associated with a significantly higher risk of IPA than COVID-19.

## Data Availability

Data are available upon request from corresponding author.
